# Crowdsourcing citation-screening in a mixed-studies systematic review: a feasibility study

**DOI:** 10.1186/s12874-021-01271-4

**Published:** 2021-04-26

**Authors:** Anna H. Noel-Storr, Patrick Redmond, Guillaume Lamé, Elisa Liberati, Sarah Kelly, Lucy Miller, Gordon Dooley, Andy Paterson, Jenni Burt

**Affiliations:** 1 Cochrane Dementia and Cognitive Improvement Group, Radcliffe Department of Medicine, University of Oxford, Oxford, OX3 9DU UK; 2 NIHR ACL in General Practice, School of Population Health & Environmental Sciences, Kings College London, London, UK; 3 Laboratoire Genie Industriel, CentraleSupélec, Université Paris-Saclay, 91190 Gif-sur-Yvette, France; 4 The Healthcare Improvement Studies Institute (THIS Institute), University of Cambridge, Cambridge Biomedical Campus, Cambridge, UK; 5 University Division of Anaesthesia at Addenbrooke’s, University of Cambridge, Cambridge, UK; 6 Metaxis Ltd, Elmbank Offices, Main Road Curbridge, Witney, Oxfordshire, OX29 7NT UK

**Keywords:** Crowdsourcing, Systematic review, Evidence synthesis, Citizen science, Information retrieval, Citations

## Abstract

**Background:**

Crowdsourcing engages the help of large numbers of people in tasks, activities or projects, usually via the internet. One application of crowdsourcing is the screening of citations for inclusion in a systematic review. There is evidence that a ‘Crowd’ of non-specialists can reliably identify quantitative studies, such as randomized controlled trials, through the assessment of study titles and abstracts. In this feasibility study, we investigated crowd performance of an online, topic-based citation-screening task, assessing titles and abstracts for inclusion in a single mixed-studies systematic review.

**Methods:**

This study was embedded within a mixed studies systematic review of maternity care, exploring the effects of training healthcare professionals in intrapartum cardiotocography. Citation-screening was undertaken via Cochrane Crowd, an online citizen science platform enabling volunteers to contribute to a range of tasks identifying evidence in health and healthcare. Contributors were recruited from users registered with Cochrane Crowd. Following completion of task-specific online training, the crowd and the review team independently screened 9546 titles and abstracts. The screening task was subsequently repeated with a new crowd following minor changes to the crowd agreement algorithm based on findings from the first screening task. We assessed the crowd decisions against the review team categorizations (the ‘gold standard’), measuring sensitivity, specificity, time and task engagement.

**Results:**

Seventy-eight crowd contributors completed the first screening task. Sensitivity (the crowd’s ability to correctly identify studies included within the review) was 84% (*N* = 42/50), and specificity (the crowd’s ability to correctly identify excluded studies) was 99% (*N* = 9373/9493). Task completion was 33 h for the crowd and 410 h for the review team; mean time to classify each record was 6.06 s for each crowd participant and 3.96 s for review team members. Replicating this task with 85 new contributors and an altered agreement algorithm found 94% sensitivity (*N* = 48/50) and 98% specificity (*N* = 9348/9493). Contributors reported positive experiences of the task.

**Conclusion:**

It might be feasible to recruit and train a crowd to accurately perform topic-based citation-screening for mixed studies systematic reviews, though resource expended on the necessary customised training required should be factored in. In the face of long review production times, crowd screening may enable a more time-efficient conduct of reviews, with minimal reduction of citation-screening accuracy, but further research is needed.

**Supplementary Information:**

The online version contains supplementary material available at 10.1186/s12874-021-01271-4.

## Background

Systematic reviews are essential to locate, appraise and synthesize the available evidence for healthcare interventions [1]. Citation-screening is a key step in the review process whereby the search results identified from searches often performed across multiple databases, are assessed based on strict inclusion and exclusion criteria. The task is performed through an assessment of a record’s title and abstract (what we term ‘citation’). The aim is to remove records that are not relevant and determine those for which the full-text paper should be obtained for further scrutiny. This is no easy task. One study found a mean of 1781 citations were retrieved in systematic review searches (ranging from 27 to 92,020 hits retrieved from searches), from which a mean of 15 studies were ultimately included in each review: an overall yield rate of only 2.94% [2]. In part driven by the resources required to undertake citation-screening, reviews are typically time and labour intensive, taking an average of 67.3 weeks to complete [2] (Borah 2017). Moreover, the challenge of locating relevant evidence for reviews is becoming ever greater: over the last decade, research output has more than doubled, and approximately 4000 health-related articles are now published every week [3–5]. New approaches are needed to support systematic review teams to manage the screening of increasing numbers of citations.

One possible solution is crowdsourcing [6]. Crowdsourcing engages the help of large numbers of people in tasks, activities or projects, usually via the internet. Such approaches have been trialled in a number of health research areas, using volunteers to process, filter, classify or categorise large amounts of research data [7, 8]. More recently, the role of crowdsourcing in systematic reviews has been explored, with citation-screening proving a feasible task for such a crowdsourced approach [9–12]. Cochrane, an international not-for-profit organization and one of the most well-known producers of systematic reviews of RCTs, is an early adopter of the use of crowdsourcing in the review process. Since the launch of their Cochrane Crowd citizen science platform in May 2016, over 18,000 people from 158 countries have contributed to the classification of over 4.5 million records [13].

To date, crowdsourcing experiments in citation-screening have often focussed on identifying studies for intervention reviews, with included studies often limited to randomised or quasi-randomized controlled trials [9–11]. Whilst this supports traditional systematic reviews concerned with evidence of effectiveness, an increasing number of reviews in health and healthcare now address research questions requiring the identification and synthesis of both quantitative and qualitative evidence [14–16]. Much less has been done to explore the effectiveness of using a crowd to screen citations for complex, mixed studies reviews. One study by Bujold and colleagues used a small crowd (*n* = 15) to help screen the search results for a review on patients with complex care needs. The study was not a validation study and so does not provide crowd accuracy measures; however, the authors concluded that crowdsourcing may have a role to play in this stage of the review production process, bringing benefit to the author team and crowd contributor alike [17].

### Aims and objectives

The aim of this feasibility study was to investigate whether a crowd could accurately and efficiently undertake citation-screening for a mixed studies systematic review. Our objectives were to assess:

1. Crowd sensitivity, determined by the crowd’s ability to correctly identify the records that were subsequently included within the review by the research team
2. Crowd specificity, determined by the crowd’s ability to correctly identify the records that were subsequently rejected by the research team
3. Crowd efficiency, determined by the speed of the crowd in undertaking the task and the proportion of records which were sent to crowd resolvers for a final decision
4. Crowd engagement, determined by qualitative assessment of their satisfaction with the citation-screening task and readiness to participate

## Methods

### The systematic review

This study was embedded within a mixed studies systematic review exploring training for healthcare professionals in intrapartum electronic fetal heart rate monitoring with cardiotocography [18]. Cardiotocography is widely used in high-risk labours to detect heart rate abnormalities which may indicate fetal distress, in order to intervene or expedite birth as required. The aim of the review was to examine the effects of training for healthcare professionals in intrapartum cardiotocography and to assess evidence for optimal methods of training. All primary empirical research studies that evaluated cardiotocography training for healthcare professionals were eligible for inclusion in the review, irrespective of study design.

### Crowdsourcing platform

**Fig. 1:**
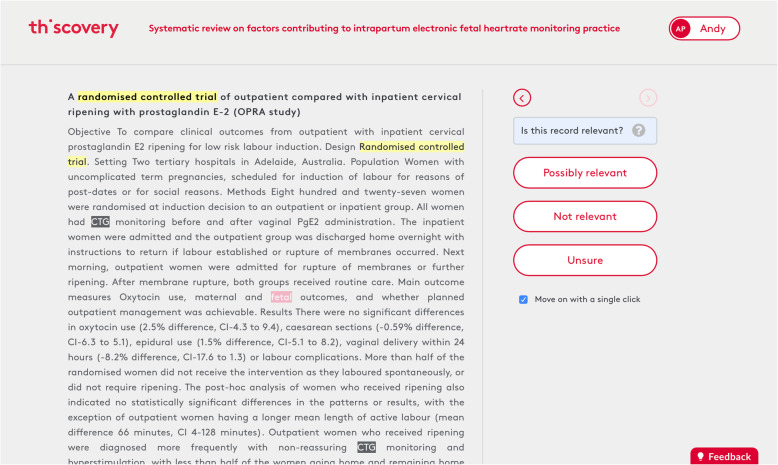
Screenshot from the task hosted on the Cochrane Crowd platform

The citation-screening task was hosted on the Cochrane Crowd platform [19]. This citizen science platform offers contributors a range of small, discrete tasks aimed at identifying and describing evidence in health and healthcare. There is no requirement for contributors to have any relevant background in research or healthcare: anyone with an interest in helping may volunteer to do so. The main activities available to contributors to Cochrane Crowd are tasks aimed at identifying or describing reports of RCTs. In these, contributors are asked to look at a series of citations (titles and abstracts of journal articles or trial registry records) and classify them as either reporting an RCT or not reporting an RCT (see Fig. 1).

Cochrane Crowd employs two strategies to ensure accuracy of contributor screening decisions. Firstly, each contributor is required to complete an interactive, bespoke customised training module prior to commencing each task, designed to improve their likelihood of making the correct classification for each record (‘individual accuracy’). Secondly, each record is reviewed and classified by multiple contributors, with an agreement algorithm used to improve the crowd’s likelihood of making the correct classification for each record (‘collective accuracy’). Typically, either three or four (depending on the task and experience of the individual screeners) consecutive identical classifications are required for a record to be labelled as either an RCT or not an RCT and removed from the screening task. Breaks in the consecutive chain, or ‘unsure’ classifications, are reviewed by crowd ‘resolvers’, highly experienced crowd contributors who make a final classification decision. Contributors are also supported in the screening task by the use of pre-specified highlighted words and phrases added automatically to each record they assess. These highlights flag notable parts of a title or abstract, and are used to direct a screener’s attention to key phrases or words which may help them make a classification decision (Fig. 1). On Cochrane Crowd, red highlights are used to flag words that may appear on citations that are unlikely to be relevant, whilst yellow highlights indicate particularly relevant keywords (such as ‘randomly assigned’, in an RCT classification task).

### Citation screening task

Citations for screening were generated through searches conducted according to the review protocol [18] (Appendix 1). The initial search identified a total of 10,223 records; after the removal of duplicates, 9546 records remained for screening.

We created two identical citation-screening tasks on the Cochrane Crowd platform: one task for the systematic review author team (*n* = 4), comprising experienced researchers undertaking the systematic review (hereafter the ‘review team’), and the second task for existing registered users on the Cochrane Crowd platform (hereafter ‘the crowd’ – see details below). The screening task presented all contributors with a series of journal titles and abstracts identified from the review searches and asked them to determine whether each record may be relevant to the topic of the review (see Fig. 1). Unlike previous tasks hosted on Cochrane Crowd, this task was a ‘topic-based’ assessment task whereby the crowd was tasked with determining the potential topic relevance of each citation rather than assessing it based on study design. Three possible classification choices were available: *Potentially relevant*, *Not relevant*, and *Unsure* (for more information on what these classification terms meant see the Task Training section below*)*. Terms for highlighting were pre-specified and based on the review search terms used (e.g. cardiotocography; training; course) and added as yellow highlights to records where they appeared. Red warning highlights were not used for this task.

### The crowd

Cochrane Crowd contributors were approached via email invitation, giving details of the review and the citation-screening task. All crowd contributors who had screened 100 or more records in Cochrane Crowd’s RCT identification task were invited to participate: this is a standard entry criterion for all more complex tasks on the platform. A ‘frequently asked questions’ document gave more detail about the study (Appendix 2). We offered a certificate of participation to all contributors, and acknowledgement in the published systematic review for those who screened 250 or more records. The Cochrane Crowd community is open to anyone with an interest in healthcare including healthcare professionals and students, researchers, patients, carers and members of the general public.

### Task training

We developed a task-specific online training module, hosted on Cochrane Crowd. This consisted of an introduction to the review topic and 20 practice records. It also included a description of the classification options available: *Possibly relevant*, *Not relevant*, and *Unsure* and guidance on when to use which option. In brief, *Possibly relevant* was to be used when records described or reported on both healthcare professional training and cardiotocography; *Not relevant* was to be used for records that were clearly not about both of one of those elements; *Unsure* was to be used if a participant was not sure either because the record contained very little information (for example a title-only record) or because the available information was simply not clear (see Appendix 3 for a pdf version of the training module). There was no pass mark for the training module and contributors could repeat the training as often as they wished. Both the crowd and the author team completed the same set of training records.

### Agreement algorithm

We used different agreement algorithms for the review team and the crowd. For the review team, we used the standard recommended algorithm for citation-screening for systematic reviews as recommended by the Cochrane Handbook [20]. Two independent contributors assessed each record and made a judgement as to whether the record was potentially relevant, not relevant or that they were unsure. Records that received discordant assessments had a final decision determined by a third member of the review team.

The agreement algorithm for the crowd required each record to be assessed by three independent contributors. Records that received discordant assessments (e.g. two *Potentially relevan*t and one *Not relevant*) were decided by a separate ‘crowd resolver’, in this case a highly experienced crowd contributor and data curation specialist selected by Cochrane Crowd (Table 1). Crowd resolvers are Cochrane Crowd contributors who have achieved exceptional accuracy on specific crowd tasks or who have extensive experience screening citations for Cochrane systematic reviews.

**Table 1:** The agreement algorithm used for the Crowd task. Breaks in the consecutive chain or any ‘unsure’ classification sends the records to resolvers to make the final decision

Decision 1	Decision 2	Decision 3	Final decision
Potentially relevant	Potentially relevant	Potentially relevant	Potentially relevant
Not relevant	Not relevant	Not relevant	Not relevant
Potentially relevant	Potentially relevant	Not relevant	Resolver decision
Potentially relevant	Not relevant	Not applicable	Resolver decision
Not relevant	Not relevant	Potentially relevant	Resolver decision
Not relevant	Potentially relevant	Not applicable	Resolver decision
Unsure	Not applicable	Not applicable	Resolver decision

### Calculating crowd sensitivity, specificity and efficiency

We calculated crowd sensitivity by identifying the proportion of citations which were subsequently included within the review by the author team and which were also correctly identified as *Potentially relevant* by the crowd (Table 2). We calculated crowd specificity by identifying the proportion of citations which were subsequently rejected from inclusion within the review by the research team and which were also rejected from inclusion by the crowd (*Not relevant*). We additionally considered crowd efficiency in terms of the speed at which the crowd completed the citation-screening task derived from the time taken for each screening classification (automatically logged by the Cochrane Crowd platform) as well as the proportion of records which were sent to crowd resolvers for a final decision.

**Table 2:** Outcome variables assessed

Outcome variable	Definition
Final sensitivity	The number of citations deemed relevant by the research team (included in the final set of studies for the review after both screening and full-text review) that were correctly identified by the crowd (true positives), divided by the number of true positives plus the number of citations included in the final set of studies by the research team that were not included by the crowd (false negative).
Screening specificity	The number of citations excluded by the crowd that were also excluded from the final set of studies by the research team (true negative), divided by the number of true negatives plus the number of citations included by the crowd that were not deemed relevant by the research team after both screening and full-text review (false positive).
Efficiency	Total time taken for the crowd versus the research team to complete the screening task.

### Replication of citation-screening task

Following completion of the citation-screening task by the crowd and assessment of the initial findings, we amended the crowd agreement algorithm to include two resolvers acting independently (rather than one resolver, as used the first round). In this replication exercise, the two resolvers each screened all records that needed resolving. Where there was a disagreement between resolver classifications (i.e. one *Potentially relevant *resolver classification and one *Not relevant* resolver classification, since resolvers could not classify a citation as Unsure), the citation was to be kept in. We then repeated the citation-screening task for the crowd, using the adjusted resolver algorithm. As before, we invited all registered users of Cochrane Crowd who had screened 100 or more records in the RCT identification task. Those who had already taken part in the first round were excluded.

### Evaluation questionnaire

In order to evaluate crowd motivations and engagement, all crowd contributors (in both the original and replication task) were asked to complete a brief online survey at the end of the task. The questionnaire covered areas including motivation to participate; experience of citation-screening; and brief socio-demographic details. Most questions were picklist-type questions but with many providing a free text option in addition. All questions were optional (Appendix 4).

## Results

### Citation-screening task

#### Contributors

Within the review team, three researchers undertook the screening task, and one researcher acted as resolver. We invited 903 Cochrane Crowd contributors to take part in citation-screening; of these, 78 (9%) participated, with 48 (62%) screening over 250 records each. An additional contributor acted as the resolver. The response rate to the post-task survey was 63/78 (81%). Fifty-one percent of respondents worked in a health-related area, 10% were patients, and 5% were carers.

#### Crowd sensitivity

**Fig. 2:**
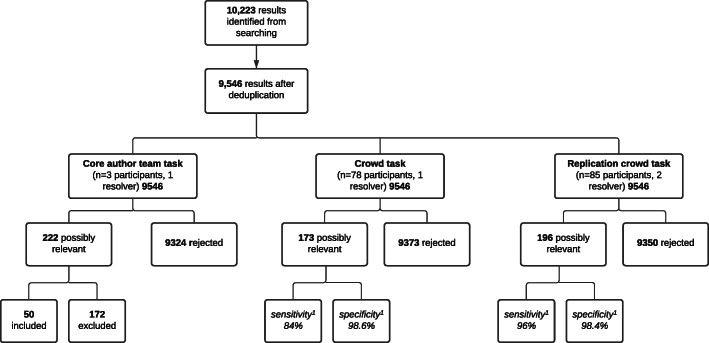
Citation screening decisions made by the review team and the crowd. ^1^Sensitivity and specificity compared to core author team as reference standard

Following citation-screening, the review team classified 222 of the 9546 records as *Potentially relevant* to the review, whilst the crowd classified 173 records as *Potentially relevant* (Fig. 2). Following full-text assessment of the 222 *Potentially relevant* citations, the review team identified 50 studies for inclusion within the review. All 50 studies had been classified by individual crowd contributors as either *Potentially relevant* or *Unsure*. However, eight of these studies which received at least one *Unsure* classification or conflicting classifications by crowd contributors were subsequently rejected by the crowd resolver. This reduced overall crowd sensitivity to 84%.

#### Crowd specificity

The review team rejected 9324 records at the citation-screening stage, and a further 172 at the full-text stage, bringing the total rejected by the author team to 9493. The crowd rejected 9373 records at the citation-screening stage, leading to a crowd specificity of 98.6%.

#### Crowd efficiency

The crowd took a total of 33 h from when the task went live on Cochrane Crowd to complete screening of the 9546 records. This included the resolution of records where the crowd had discordant classifications or had classified a record as *Unsure*. The review team took a total of 410 h to complete screening. However, crowd contributors took longer on average to screen an individual record compared to a member of the core author team (mean of 6.06 s per record for the crowd compared to 3.97 s per record for the core author team). For the crowd task, 677 (7.09%) records needed resolving; in the review team task 420 (4.39%) records needed to be resolved.

#### Replication of citation-screening task

The citation-screening task was replicated to assess consistency of crowd performance, and to evaluate a different agreement algorithm whereby two resolvers rather than one resolver assessed all records that needed resolving. This was because in the original task, resolver error had led to a reduction in crowd sensitivity.

**Fig. 3:**
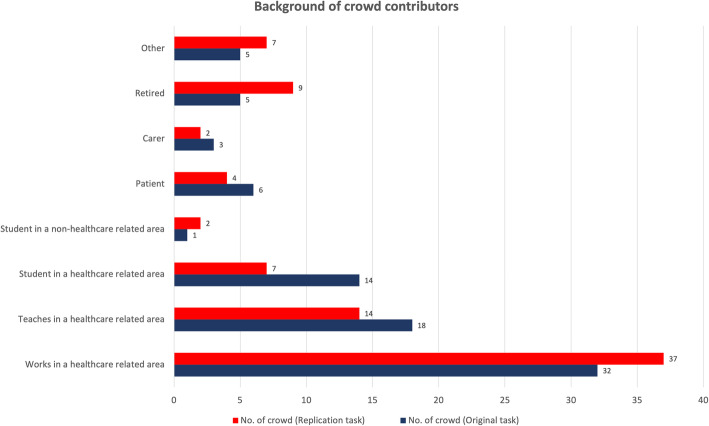
Clustered bar chart showing crowd contributor backgrounds for original and replication tasks. 63 out of 78 (81%) participants completed the survey for the original task; 64 out of 85 (75%) participants completed the survey for the replication task

Eighty-five participants contributed to the replication task. None of the 85 contributors for this task had taken part in the original task. There was little variation in the background of contributors in the replication study compared to the original contributors (Fig. 3). The response rate to the post-task survey for the replication task was 64/85 (75%). Of those responders, 58% of respondents worked in a health-related area, 6% were patients, and 3% percent were carers.

The crowd took 48 h to complete the second citation-screening task. 889 (9.3%) records of the 9546 screened were referred to the crowd resolvers, either due to discordant or *Unsure* classifications. No included study referred to the resolvers was subsequently rejected. Two previously included studies were however rejected by the crowd during the replicated task. Crowd sensitivity improved from 84% in the original study to 96% (48 out of the 50 studies correctly classified as *Potentially relevant*) in the replication study. The crowd’s specificity in the replication task was similar to the original value, at 98.4% in the repeated study compared to 98.6% in the original study.

#### Crowd engagement

The primary motivation to participate amongst respondents to the questionnaire was ‘to do something for a well-respected organisation’. This was followed by the chance to get acknowledgement on a review. 97% of respondents reported enjoying the task in both the original and replication task surveys, and a similar proportion, again for both surveys, said that they found the task either easy or very easy (84% for the original task and 90% for the replication task). None of the respondents reported that they found the task difficult.

When asked whether they preferred the usual RCT identification task available in Cochrane Crowd or the new topic-focused task, the responses were evenly split between preferring the new topic-focused task or liking both tasks. Only 10% reported that they definitely preferred the RCT identification task (for the replication task, only 6% preferred the RCT task).

When asked about the use of highlighted words and phrases, 92% of respondents felt that they had been useful. This was also the case for the replicated task where 89% of respondents felt they had found them useful. At the end of the survey people had the chance to make any further comments. Thirty-six people made comments, with the vast majority reflecting their enjoyment of the task or the satisfaction of being involved in the feasibility study:

“I thought this was an excellent pilot project. If these were offered more frequently, I would assign my students to participate”

“I think it is a very useful way to spend half an hour when I have the spare time, it made me feel connected, and it seemed to achieve a lot for the review”

“Please do more. Please keep doing this. I feel much more connected to things, being offered a role however small in somebody’s research, I value this immensely.”

“Was good to have a smaller task on offer as it felt more ‘doable’ and that my contribution would really make a difference”

## Discussion

In this feasibility study examining the potential for crowdsourcing citation-screening in a mixed studies systematic review, crowd contributors correctly identified between 84% and 96% of citations included in the completed review, and 99% of citations which were not included. On both occasions, citation-screening was completed by the crowd in 2 days or less. These results compare very favourably to other studies exploring topic-based crowd citation-screening and time outcomes [9, 11, 21] though direct comparisons are difficult due to the variation in review types and tasks being evaluated.

Whilst the sensitivity and specificity of the crowd appear high, there were misclassifications made by both contributors and resolvers when compared to the decisions of the review team. In the first citation-screening task, eight studies identified as potentially relevant by the crowd, and included in the review by the review team, were later rejected by the crowd resolver. In the replication task, the crowd collectively rejected two studies included in the review by the review team. With a strengthening of the resolver function in the replication task (with two resolvers working independently, rather than one resolver), no included studies that needed resolving were rejected, suggesting crowd sensitivity was boosted by the use of a more robust agreement algorithm. In part, this may be due to a decreased risk of screening fatigue with more than one resolver available to adjudicate screening disagreements or uncertainties [22] but also, as two recent studies have confirmed, a single screener (versus dual screening) is likely to miss includable studies [23, 24].

Both of the studies rejected by the crowd in the replication task [25, 26] were amongst the eight rejected by the resolver in the first round of the study. Rejection of these papers by the crowd may have been influenced by the highlighting of words and phrases in the records. The record for Blomberg 2016, contained only one highlighted word (‘training’), whilst the Byford 2014 paper contained no highlighted words. In comparison, the records for other studies identified through screening tended to contain a larger number of highlighted words.

The relative speed of the crowd in completing the citation-screening was similar to previous tasks undertaken by Cochrane Crowd. In a series of pilot studies run in July 2017, contributors were tasked with screening search results for four Cochrane reviews. The number of results to screen within each review ranged from approximately 1000 to 6000: the mean time taken by the crowd to complete citation-screening was 24 hours [27]. Such speed enables a review team to move rapidly from search results to full-text screening: an advantage when the time taken to complete many reviews means searches have to be updated and further screening and data extraction undertaken prior to publication. Whilst the increased speed of screening raises the potential for time and cost savings through using crowdsourcing, this does not account for the time taken to design, build and pilot the training and instructions for each review. This training was ‘bespoke’, customised to the review, marking a departure from the RCT identification tasks normally hosted on Cochrane Crowd, and thus more resource intensive to develop. Therefore, the trade-off between speed of crowd screening and resources to enable crowd screening needs further scrutiny. With searches for mixed studies reviews often generating very high numbers of search results to assess, the time spent creating customised topic-based training modules might be well justified.

An alternative approach to crowd citation-screening might be to reframe the nature of the crowd screening task itself. This study, like others before it, asked the crowd to screen search results against the same criteria used by the core author team. This provides good comparative data for crowd performance calculations. However, a more effective approach may be to ask crowd screeners to focus on the identification of very off-topic citations, changing the overarching question from “*Does the record look potentially relevant?”* to *“Is the record obviously not relevant?”.* Approaching crowd tasks for complex reviews in this way might make the compilation of the training module less time and resource intensive, as well as improving crowd sensitivity. The obvious detrimental impact would be on specificity, as a greater proportion of irrelevant records would be kept in. However, following ‘first pass screening’ from crowd contributors, author teams would be able to undertake title-abstract screening of a substantially smaller number of remaining records with a higher prevalence of potentially relevant records. To our knowledge, this approach has not been explored.

Another approach may be to explore the role of machine learning in combination with crowd effort. Machine learning classifiers are being used increasingly to help identify RCTs and other study designs [28–31]. Within a mixed studies context, the main challenge would be generating enough high-quality training data for the machine. However, for searches that retrieve a high volume of hits it may be feasible to build a machine learning model from a portion of crowd- or author-screened records, that could then either help to prioritise/rank remaining records by likelihood of relevance or be calibrated at a safe cut-off point to automatically remove the very low-scoring records.

Our findings are inevitably limited by their dependence on citation-screening of search results from only one systematic review. Different search results from different reviews may generate different sensitivity and specificity estimates in crowdsourced citation-screening. However, it is notable that there is little published evidence on how screening decisions vary: different expert review teams could also be anticipated to make different screening decisions when presented with the same set of search results. With a complex mixed studies review, the likelihood of human error, whether from the crowd or the ‘expert’ review team, is further increased: there is often limited information in abstracts to judge topical relevance. It is not clear what an acceptable level of crowd accuracy is, to be able to confidently use crowdsourcing without comparing crowd decisions to those of an expert review team. For reviews of evidence of effectiveness, there may be very little tolerance for divergence of decisions. For other reviews – such as the current example on training in the use of cardiotocography – overall review results may be little influenced by the inclusion or exclusion of a few studies of marginal quality and depth of information. The level of error deemed to be acceptable in relation to a specific degree of time saving may depend on both the type of review being conducted and the breadth and volume of potentially includable studies. These are factors that require determining if crowdsourcing in this way is to become an acceptable model of research contribution.

Finally, In terms of the generalisability of these results we should address the characteristics of the crowd participants. Whilst we recruited a non-selective crowd (contributors did not need to have any topic knowledge or expertise to be able to participate) we can see from the survey responses that many participants did have a healthcare background which may have made the task easier. In addition, in order to be able to participate in this study, potential participants had to have completed 100 assessments in another Cochrane Crowd task, RCT Identification. The RCT Identification task on Cochrane Crowd requires contributed to complete a brief training module made up of 20 practice records. While this task is different from the study task, it does mean that the participants were already familiar with screening citations within Cochrane Crowd. We therefore must exercise caution in generalising that a crowd consisting of either fewer healthcare professionals or those without any experience of screening citations, would perform as successfully.

Finally, the very positive responses from this study’s participants were highly encouraging. However, successful, widespread implementation of crowdsourcing in this way brings with it a number of important ethical considerations. Providing meaningful opportunities for people to get involved with the research process must be matched by appropriate measures of acknowledgement and reward. In this study named acknowledgement in the review proved a suitable reward but as crowdsourced tasks become more involving or challenging, as they no doubt will, it stands that the requisite reward should be greater. This then potentially presents a conflict with current academic publishing guidance, with criteria for authorship often requiring full involvement of all authors across all or many parts of the study. In some circumstances, payment might be appropriate, yet micro-payment or piece-rate models such as those used by Amazon Mechanical Turk have come under fire in recent years with studies revealing poor working conditions of an “unrecognised labour” [32, 33]. As crowdsourcing in this way becomes more accepted as an accurate and efficient method of study identification, these ethical factors will need to be understood and addressed in parallel, for the benefit of both contributor and task proposer alike.

## Conclusion

In support of a complex mixed-studies systematic review, a non-specialist crowd tasked with undertaking citation-screening performed well in terms of both accuracy and efficiency measures. Importantly, crowd members reported that they enjoyed being part of the review production process.

Further research is required to develop effective approaches to pre-task training for contributors to crowd-sourced citation-screening projects, the refinement of agreement algorithms, and establishing ‘acceptable’ levels of performance (for example, by investigating the variation in performance by both crowd and ‘expert’ screening teams, such as clinicians).

Review teams, particularly those engaged in locating a broad range of evidence types, face significant challenges from information overload and long production times. With further refinements in its approach, crowdsourcing may offer significant advantages in terms of time-saving, building capacity, engagement with the wider evidence community and beyond, with a minimal loss to quality.

## Supplementary Information

**Additional file 1.**

**Additional file 2.**

**Additional file 3.**

**Additional file 4.**

**Additional file 5.**

## Data Availability

The data used and/or analysed during the current study are included in this published article (Appendix 5).
