# Supplementary material for: Crowdsourcing citation-screening in a mixed-studies systematic review: a feasibility study
Source: BMC Med Res Methodol. 2021 Apr 26;21:88. doi: 10.1186/s12874-021-01271-4 (PMC8077753; doi:10.1186/s12874-021-01271-4)
Supplement: Supplementary file 1 — Additional file 1. [file 12874_2021_1271_MOESM1_ESM.pdf]

NHS
National Institute for Health Research

Health professional training for cardiotocography interpretation and management: protocol for a systematic review of quantitative and qualitative evidence

Patrick Redmond, Guillaume Lame, Elisa Liberati, Rebecca Simmons, Jenni Burt, Mary Dixon-Woods

Citation

Patrick Redmond, Guillaume Lame, Elisa Liberati, Rebecca Simmons, Jenni Burt, Mary Dixon-Woods. Health professional training for cardiotocography interpretation and management: protocol for a systematic review of quantitative and qualitative evidence. PROSPERO 2018 CRD42018082567 Available from:
http://www.crd.york.ac.uk/PROSPERO/display_record.php?ID=CRD42018082567

Review question

To assess the effects of training healthcare professionals to interpret and act on cardiotocography traces. We will seek to characterise the training approaches used, and consider the effects of these training approaches on knowledge, skills, attitudes, and behaviours of healthcare professionals; patient outcomes; and resource use.

Searches

The search strategy, written in conjunction with a medical librarian, will include the following databases (Appendix 1).

Electronic searches

• Cochrane Central Register of Controlled Trials (CENTRAL), The Cochrane Library

• MEDLINE

• Embase

• PsycINFO

• British Nursing Database, ProQuest

• Cumulative Index to Nursing and Allied Health Literature (CINAHL)

• ERIC, Institute of Education Sciences

• Dissertations and Theses Database, ProQuest

• Scopus

• Web of Science, Clarivate Analytics

We will translate the MEDLINE search strategy for other databases using appropriate syntax and vocabulary for those databases. The strategy includes medical subject headings and synonyms for CTG and training.

Searching other resources

We will conduct a grey literature search to identify studies not indexed in the databases listed above. Sources will include the sites listed below:

• OpenGrey

• Grey Literature Report

• NICE Evidence Search

We will search the following registries:

• International Clinical Trials Registry Platform (ICTRP)) search portal, WHO

• ClinicalTrials.gov, US National Institute of Health (NIH)

We will also:

• review reference lists of all included studies, and those of relevant systematic reviews;

• contact authors of relevant studies or reviews to clarify reported published information/seek unpublished results/data;

• contact researchers with expertise relevant to the review topic

Types of study to be included

Our objectives require consideration of the fullest range of evidence to enable a characterisation of CTG training approaches developed to date, and their impact on the four Kirkpatrick levels (reactions; learning; behaviours; and results). Study designs will be categorised into one of three groups:

1. Randomised controlled trials

2. Observational studies with a control group, including:

a. controlled before after studies;

b. interrupted time series studies;

c. repeated measures studies;

d. cohort studies (with participants acting as their own controls);

e. simple pre-post studies;

f. case control studies.

3. All other study designs, including:

a. Observational studies without a control group

b. Quality improvement reports

c. Surveys

d. Qualitative research

We will restrict qualitative studies to those reporting empirical data collection and analysis (K Hannes, 2011). Studies will be eligible for inclusion irrespective of language or publication status (for example, conference abstracts will be included).

Condition or domain being studied

Intrapartum monitoring of the foetal heart rate via CardioTocoGraphy is used extensively in high-income countries (Parer, 2003b; Ugwumadu et al., 2016; Wheble et al., 1989). CTG is one of two methods to monitor foetal heart rate during labour (the other being intermittent auscultation via Doppler ultrasound). Continuous CTG monitors both the foetus's heart rate and the woman's uterine contractions. The recordings are graphed

and the interpretation of anomalies by healthcare professionals triggers appropriate action (American College of Nurse-Midwives, 2015). The National Institute for Health and Care Excellence (NICE) and the American College of Obstetricians and Gynaecologists both recommend its use, with NICE recommending CTG for high-risk pregnancies and intermittent auscultation for low-risk pregnancies (NICE 2014; ACOG 2010).

Interpreting CTG traces is complex. Four features of the graph are used for interpretation:

- the baseline heart rate

- the baseline variability

- the presence of accelerations, and

- the presence or absence of early or late decelerations

The NICE guidelines provide criteria to classify each feature as “reassuring”, “non-reassuring” or “abnormal”. Based on the classification of the features, the graph is classified in one of four ways:

1. “normal” (all features are reassuring)

2. “suspicious” (one non-reassuring feature and two reassuring features)

3. “pathological” (one abnormal feature or two non-reassuring features)

4. “need for urgent intervention” (acute bradycardia, or a single prolonged deceleration for 3 minutes or more)

NICE guidelines provide management indications for each situation. They also recommend documenting the woman’s condition and CTG interpretation every hour, and seeking senior advice (from a senior midwife or an obstetrician) when the CTG is difficult to interpret.

Why it is important to do this review

Preventable harm related to childbirth has far-reaching consequences for both the families and caregivers involved (Kirkup 2015). Maternity safety is variable, with a higher perinatal mortality rate reported in England compared to the rest of the UK (National Audit Office 2013). It is also costly; obstetric claims are disproportionately represented in NHS resolution payments (£1,921 million in 2016/2017) (NHS Resolution 2017). Errors in foetal monitoring occupy a major source of risk in maternity care, with alleged CTG misinterpretation representing 15% of the estimated total value of claims between 2000 and 2010 for the NHS Litigation Authority (NHS Litigation Authority 2012).

Studies consistently show considerable levels of inter- and intra-observer variability in the interpretation of CTGs (Chauhan 2008; Figueras 2005; Palomaki 2006; Sabiani 2015). Broad consensus exists on the need for high-level technical proficiency in CTG interpretation; lack of skills in how to interpret and react to CTGs based on established guidelines is regarded as a leading cause of CTG-related incidents in maternity care (NHS Litigation Authority 2012; RCOG 2017). However, the optimum format for instruction, the number of hours, frequency of training and testing, as well as the validity of testing methods is unknown (Ugwumadu 2016). In sum, much uncertainty remains on what type of interventions may prevent inadequate interpretation of, and response to, CTGs.

A previous systematic review on CTG training (Pehrson 2011) found that training interventions were highly heterogeneous in format and content (including e-learning, case reviews, monthly audit with feedback, voluntary review sessions, and clinical supervision through tele-didactics). The review presented findings narratively for all study designs; whilst six randomised studies were included within the review, results from these were not considered separately to all other study designs. Consequently, it is difficult to draw definitive

conclusions on what features and mechanisms of training are linked with the acquisition of new knowledge, the adoption of new behaviours, and the impact on outcomes. It is also challenging to untangle the level of current evidence associated with different training interventions. The authors of the systematic review also noted that the generally poor quality of many of the reviewed studies warrants caution with the findings (Pehrson 2011). Since the publication of the Pehrson review, further evaluations of CTG training (including randomized studies) have been completed (Carbonne 2016; Millde-Luthander 2012; Byford 2014). It is therefore timely to repeat, update and strengthen the previous review in this field.

Participants/population

We will include all studies involving healthcare professionals receiving CTG training. These may include, but are not limited to, medical students, midwives, and practising clinicians. There will be no restriction on profession, location, or educational attainment.

Intervention(s), exposure(s)

We will include all studies describing or exploring the effect of training healthcare professionals to interpret and act on cardiotocography traces.

This includes studies describing or exploring the effect of training healthcare professionals to interpret and act on cardiotocography traces within a more complex or wider intervention (for example, studies that utilise two or more interventions aimed at changing health professionals' learning or behaviour), provided a discrete analysis of the CTG component is provided.

We will apply no restrictions on the basis of the length of training; we will include short programmes such as single lectures, workshops, and modules, as well as more extended educational programmes. We will apply no restrictions on the basis of the mode/s in which training is delivered, which may include e-learning, workshops, case studies, lectures, role-play, and computerised decision support.

Comparator(s)/control

If applicable, the comparator group will be those participants receiving no intervention or “usual care”, as determined by the specific health care environment or training.

Context

There are no restrictions in terms of settings (e.g. geographical, health system). However, it is likely most studies will report findings from training delivered to individuals either intending to or actually providing care to pregnant women.

Main outcome(s)

We will include studies reporting on a range of outcomes in line with the relevant literature. In keeping with the objective to update the existing review, the Kirkpatrick four level model of training evaluation will be used to categorise the outcomes in line with the level of evaluation they represent (Kirkpatrick 1998):

• Level 1: Reactions. Participants’ reactions to and opinions about the training

• Level 2: Learning. The extent to which participants have acquired new knowledge, skills or attitudes

• Level 3: Behaviour. The extent to which learning is applied in the workplace

• Level 4: Results. The wider impact of the learning (such as changes in resource use or patient outcomes)

Due to the broad nature of the review, and the diverse study designs and evidence sources we are likely to locate, we have not pre-specified detailed primary and secondary outcomes. Instead, we outline four areas in which we will seek to analyse and synthesise the available evidence:

1. CTG classification outcomes

2. Healthcare provider outcomes

3. Clinical/Patient outcomes

4. Additional outcomes

Additional outcome(s)

None

Data extraction (selection and coding)

A combination of two authors will independently screen titles and abstracts to decide which studies satisfy the inclusion criteria, as well as identifying multiple reports from single studies. Any papers not meeting the inclusion criteria will be excluded at this stage. If there is uncertainty, consensus will be reached by discussion with another co-author. Following this, two authors will independently assess the full text articles to ensure studies still fulfil the inclusion criteria.

Data extraction and management

A combination of two authors will independently undertake data extraction using a predefined data collection checklists, including Kirkpatrick's four-level model to assess outcomes. Data on the development, design, methods, context, and the populations involved will be extracted in a standardised way, using an established tool designed for a broad range of study types in the education sphere (EPPI Centre, n.d.). Any disagreement will be resolved by discussion between co-authors. Where necessary, we will contact authors for missing information or clarification. Where relevant, information from data extraction forms will guide the extraction of numerical data for possible meta-analysis.

Risk of bias (quality) assessment

The Joanna Briggs Institute critical appraisal tools will be used to assess the risk of bias and quality in both quantitative and qualitative studies (The Joanna Briggs Institute, n.d.). These tools include domains such as appropriateness of the research design to meet the stated aims, rigour of data-collection and analysis, well-conducted and accurate sampling strategy, clear statements of findings, accurate representation of participants' voices, outline of the researchers' potential influences, background, assumptions, justifications of the conclusion or whether or not it flows from the data, as well as value and transferability of the research project.

A combination of two authors will independently perform the quality assessment. We will resolve disagreements by discussion and, if needed, arbitration by a third author. We will present quality assessments in tabular format. For each study, we will provide a summary assessment of overall study quality as follows:

• Low quality

• Medium quality

• High quality (satisfying all criteria)

Studies reported as low quality will not automatically be excluded; instead, their exclusion will be examined through sensitivity analysis (Dixon-Woods et al., 2007).

Assessment of reporting biases

Where relevant, we will examine asymmetry in funnel plots of the primary outcome to assess the potential for study effects such as publication bias, if a sufficient number of trials are available. We will conduct formal statistical tests for funnel plot asymmetry, namely the Begg's and Egger's methods (Higgins and Green, 2011), again if a sufficient number of trials are available. Furthermore, we will assess reporting bias by scrutinising the study results using the 'Risk of bias' tables. Where there is a possibility of publication bias and small-study effects, we will undertake a sensitivity analysis as described. In addition to searching trial registries for relevant trials not identified in our main database searches, we will also search for protocols of studies selected for inclusion, to compare planned with actual methods, interventions and outcomes.

Furthermore, a thorough search of the grey literature and contact with known experts in the field will also reduce the influence of publication bias on our review.

Strategy for data synthesis

Quantitative and qualitative evidence analysis and syntheses

Quantitative evidence

Where relevant, we will report outcomes for each study in natural units. We will calculate, where possible, absolute change from baseline with 95% confidence intervals. We will report estimates for dichotomous outcomes (e.g. incorrect CTG trace classification) as risk ratios. We will report estimates for continuous outcomes as mean differences if they are measured on the same scale; if continuous outcomes are measured on multiple scales, we will report the standardised mean difference. We will report pre-intervention and post-intervention means or proportions where baseline results are available for both intervention and control groups.

We will combine findings from independent studies using standard meta-analysis techniques, provided enough study data is obtained and taking account of heterogeneity between studies. The size of the study will determine the study’s weight and an overall treatment effect will be estimated.

For interrupted time series design studies, we will extract the difference in slope and the difference in pre to post-intervention levels. We will analyse the post- versus pre-intervention difference (adjusted for trends) at specific time points (three months, six months and six-monthly thereafter). If the differences are not available in the primary reports, we will attempt re-analysis using data from graphs or tables

Re-analysis, to estimate the effect of an intervention, will include a segmented time-series regression analysis, taking into account secular time trends and any autocorrelation between any individuals observations. This allows the change in level and change in trend, after the intervention, to be estimated. Meta-analysis will be performed for the changes in level and changes in trend using the generic inverse variance method.

We will tabulate all relevant information of studies included in the review. This will include all pre- and post-intervention results (sample sizes, means, proportions, 95% confidence intervals, etc.) for each group for each outcome of interest. Additionally, we will examine the pre- and post-intervention difference for each group for each outcome of interest as well as the differences across groups. We will conduct a meta-analysis combining the results of the individual studies.

If we identify sufficient studies with quantitative data we will perform statistical analysis using review management software. We will conduct the meta-analysis of included randomised controlled trials and observational trials both together and separately (see Sensitivity analysis). Pooled estimates (risk ratios (RRs) with 95% confidence intervals (CIs)) of the evaluated outcome measures will be calculated by the generic inverse variance method. Results will not be depicted as ’not statistically significant’ or ’non-significant’, but we will report the CIs together with the exact P value. In the absence of statistical and clinical heterogeneity we will apply a fixed-effect model to pooled data. The I^2 statistic will be examined to describe the proportion of the variability in the results that reflects real differences in effect size. However, variation in studies with respect to populations, interventions, outcomes and settings is likely. Thus, the true effect is likely to be related, but not the same for all studies. We will therefore choose the random effects model or choose not to perform a meta-analysis.

Assessment of heterogeneity

We intend to assess contextual heterogeneity on the basis of information collected on the context in which the intervention was implemented. We will assess for variability in the participants, interventions and outcomes studied to identify clinical heterogeneity. Statistical heterogeneity will be identified and measured as recommended by the Cochrane Handbook for Systematic Reviews of Interventions (Higgins 2011). The following will be used as a guide for interpretation:

• 0% to 40%: might not be important;

• 30% to 60%: may represent moderate heterogeneity;

• 50% to 90%: may represent substantial heterogeneity;

• 75% to 100%: considerable heterogeneity.

Unit of analysis issues

Cluster-randomised trials selected for inclusion will be assessed in order to ensure that appropriate analysis was carried out to address cluster effects and to avoid overestimating the significance of differences. In cluster randomised studies where the analysis was carried out ignoring the effect of clustering, efforts will be made to obtain the data needed to correct for this. Should the data not be forthcoming we will use the intra cluster correlation coefficient (ICC) or design effect from external sources (other trials included in the review) to inflate the standard error.

Qualitative evidence

Using an established meta-aggregative qualitative approach, we will interrogate and synthesise findings from qualitative studies, using the following steps (Godfrey and Harrison, 2015):

• Step 1: Extraction of all findings

• Step 2: Grouping findings into categories

• Step 3: Grouping categories into synthesized findings

In Step 1, we will assemble findings from all included qualitative studies, provide illustrations of these findings, and assign a level of credibility to these findings. Levels of credibility have been previously defined as (Pearson, 2004):

• Unequivocal (findings are accompanied by a robust illustration which is sufficient to prevent challenge)

• Plausible (findings are accompanied by an illustration which lacks clear associations with these, and are therefore open to challenge)

• Unsupported (findings are not supported by the data)

In Step 2, we will develop categories for these findings, developed on the basis of similarity in meaning.

Finally, in Step 3, we will develop a comprehensive set of synthesized findings, bringing together two or more categories of findings to present an aggregated framework describing the available evidence. Aggregation will be facilitated by the use of review management software.

Convergent data synthesis of quantitative and qualitative evidence

To facilitate a comprehensive assessment of the effects of training healthcare professionals to interpret and act on CTG traces, we will seek to bring together quantitative and qualitative evidence to generate a full description of CTG training programmes and their impacts. To achieve this, we will use a results-based convergent synthesis design. (Hong, Pluye, Bujold, & Wassef, 2017; The Joanna Briggs Institute Reviewers' Manual, 2014). We will initially conduct separate syntheses of available quantitative and qualitative evidence, as described in detail above. Once completed, we will undertake a secondary synthesis, using (as appropriate) the pooled qualitative findings as a sensitising framework with which to further examine the synthesised quantitative evidence, and/or bringing together statements synthesising the quantitative evidence with those from the qualitative evidence. The precise approach to data integration will be decided following completion of initial, separate, quantitative and qualitative evidence syntheses; if possible, we will include a mixed methods matrix approach in which aggregated findings are compared (O'Cathain, Murphy, and Nicholl, 2010). Separate matrices will be developed using Kirkpatrick's four-level

framework, to enable interrogation of the available evidence for the impact of training across these different areas. This final meta-aggregative process will enable us to generate a comprehensive categorization of all findings across all study types (Karin Hannes and Lockwood, 2011).

Summary of findings table

We will conduct quality assessment of the results using the Grading of Recommendations Assessment, Development and Evaluation Confidence in the Evidence from Reviews of Qualitative research (GRADE – CERQual) approach, which specifies four levels of quality (high, moderate, low and very low) where the finding is rated on methodological, relevance, coherence and adequacy of the data (Lewin et al., 2015). Quality will be assessed separately for each finding.

Analysis of subgroups or subsets

Subgroup analysis

We will pursue further subgroup analyses with sufficient data. Exploratory search results suggest the following subgroup analysis may be possible.

• Intervention type (e.g. e-Learning, workshops, clinical setting decision aids)

• Population (midwives, medical students, clinicians)

• Setting (regulatory environment - formally accredited versus non accredited training programme)

The number of subgroups will be kept to a minimum and priority will be given to subgroups that are of specific interest to the potential implementation of any future intervention.

Sensitivity analysis

We will conduct a sensitivity analysis to calculate the effect of risk of bias/quality (including missing data) within studies on effect size, by calculating the effect of excluding or including studies with a higher risk of bias. To determine if the methodologic quality of study design will affect the findings, sensitivity analyses will be conducted restricting pooling of studies to those of higher methodologic quality such as RCTs only.

Contact details for further information

Patrick Redmond
pr440@medschl.cam.ac.uk

Organisational affiliation of the review

The Healthcare Improvement Studies Institute
https://www.thisinstitute.cam.ac.uk/

Review team members and their organisational affiliations

Dr Patrick Redmond. THIS Institute (The Healthcare Improvement Studies Institute)
Dr Guillaume Lame. THIS Institute (The Healthcare Improvement Studies Institute)
Dr Elisa Liberati. THIS Institute (The Healthcare Improvement Studies Institute)
Dr Rebecca Simmons. THIS Institute (The Healthcare Improvement Studies Institute)
Dr Jenni Burt. THIS Institute (The Healthcare Improvement Studies Institute)
Professor Mary Dixon-Woods. THIS Institute (The Healthcare Improvement Studies Institute)

Anticipated or actual start date

13 February 2018

Anticipated completion date

01 June 2018

Funding sources/sponsors

The Health Foundation funds The Healthcare Improvement Studies Institute (THIS) at the University of

Cambridge. The review takes place as part of a body of work examining the safety of foetal monitoring and interventions to improve training of healthcare professionals involved in the delivery of care to women during labour.

Conflicts of interest

Language
English

Country
England

Stage of review
Review_Ongoing

Subject index terms status
Subject indexing assigned by CRD

Subject index terms
Attitude; Cardiotocography; Female; Health Personnel; Health Resources; Humans; Knowledge; Pregnancy

Date of registration in PROSPERO
12 February 2018

Date of publication of this version
23 May 2018

Details of any existing review of the same topic by the same authors

Stage of review at time of this submission

Stage	Started	Completed
Preliminary searches	Yes	Yes
Piloting of the study selection process	No	No
Formal screening of search results against eligibility criteria	No	No
Data extraction	No	No
Risk of bias (quality) assessment	No	No
Data analysis	No	No

Versions
12 February 2018
23 May 2018

PROSPERO
This information has been provided by the named contact for this review. CRD has accepted this information in good faith and registered the review in PROSPERO. CRD bears no responsibility or liability for the content of this registration record, any associated files or external websites.
