# Supplementary material for: Crowdsourcing citation-screening in a mixed-studies systematic review: a feasibility study
Source: BMC Med Res Methodol. 2021 Apr 26;21:88. doi: 10.1186/s12874-021-01271-4 (PMC8077753; doi:10.1186/s12874-021-01271-4)
Supplement: Supplementary file 2 — Additional file 2. [file 12874_2021_1271_MOESM2_ESM.pdf]

Help find research about cardiotocography monitoring
Frequently asked questions

What is this task?
This is a task aimed at a specific review: **Health professional training for cardiotocography interpretation and management**. Unlike previous tasks, we're not asking you find randomised trials (RCTs) – not yet anyway! For this task we want you to find records that seem relevant to this review, whether they are RCTs or not. This is new territory for us! We have never posted a task where the question is: *does this record look relevant*? It's going to be really interesting to see how it goes.

But I don't know anything about cardiotocography – does that matter?
Don't worry if you are new to cardiotocography and fetal heart monitoring. We've put together a brief, interactive training module. It won't make you an expert in cardiotocography but it should give you the information you need to perform this task well.

Why are we doing this?
For this task **we're teaming up with The Healthcare Improvement Studies Institute (THIS Institute)**. This is a new institute based at the University of Cambridge; it launched in January this year with a mission to strengthen the evidence base for improving the delivery of healthcare in the NHS. With a highly collaborative approach, working with patients and NHS staff, THIS Institute will conduct research that is of relevance to those audiences. Their ethos is closely aligned with the Cochrane mission to improve the evidence used in healthcare decision making, and we are really excited to be working together.

This is the first time we've done this and we're viewing this task as a pilot exercise where we hope to gain a better understanding about how well this kind of task goes and how Cochrane Crowd and the Institute might be able to collaborate in the future. So, THIS Institute researchers will compare the screening judgments of Cochrane Crowd to their traditionally conducted review. Exciting!

Can anyone join this task?
Actually no. We have specifically given you access to this task because you have screened 200 or more records in Cochrane Crowd's mainstream RCT identification task.

Do I have to do this task instead of my usual Cochrane Crowd task?
If this task doesn't appeal to you, you can just ignore it and carry on with the main Cochrane Crowd tasks.

How long will this task be around for?
This task will be live for two weeks. There are about 10,000 records to go through so hopefully two weeks will be enough time to get through the lot. If not, we'll extend the deadline a bit.

What do I get out of this?
If you screen 250 or more records as part of this task, you will be acknowledged by name in the review when it is published. However, this is entirely voluntary and you will be asked in advance by Cochrane Crowd whether you would like to be identified. THIS Institute anticipate completing their review by the end of 2018. The review is not a Cochrane review but it will be a very high quality review in an important area for healthcare improvement.

What information will be shared with THIS institute?
Firstly, as you know we would never share any information that will identify you without getting explicit consent first. For instance, we will contact you if you qualify for acknowledgement for this task to confirm if you wish to be acknowledged and for your permission to pass your name on to THIS institute - they would only use your name for the purpose of acknowledgement in publication.

To get the best understanding of whether this task is successful and how a collaboration might work with THIS institute in future, we will also share fully anonymized information on your feedback of the task (see below for more on this), and metrics around number of citations screened, time taken, classifications made, number/proportion of disagreeing classifications requiring resolution, and prior experience with Cochrane Crowd.

Can I feedback my thoughts?
We will be sending out a brief survey at the end of this pilot exercise where you will be able to feedback your thoughts. However, if you have any questions, queries or suggestions in the meantime please don't hesitate to email either Anna or Emily at crowd@cochrane.org.
