# Supplementary material for: Crowdsourcing citation-screening in a mixed-studies systematic review: a feasibility study
Source: BMC Med Res Methodol. 2021 Apr 26;21:88. doi: 10.1186/s12874-021-01271-4 (PMC8077753; doi:10.1186/s12874-021-01271-4)
Supplement: Supplementary file 3 — Additional file 3. [file 12874_2021_1271_MOESM3_ESM.pdf]

**Training module for THIS pilot task 1 – FINAL**
+++++++++++++++++++++++++++++++++++++++++

This training module is made up of some introductory screens and 10 practice records. It should take about 20 minutes to complete. By the end of it you will be able to progress to the live task.

The task is all about identifying records for a specific systematic review.

The review is called: **Health professional training for cardiotocography interpretation and management.**

Unlike previous Cochrane Crowd tasks, this task is not going to ask: *Is the record describing an RCT?* The question here is: *Does this record look like it might be relevant to this review?*

Shall we begin?

[NEXT]
++++++++++++++++++++++++++++++++++++++++++++++++
Okay, so let's go back to the review title:

## Health professional training for cardiotocography interpretation and management

It's a little bit of a mouthful, but then review titles often are as they try to convey the key elements of the topic that the review is going to cover.

Which **two words** in the title do you think might be the most important for a task that aims to identify any possibly relevant records without throwing out (or rejecting) any that are relevant?

[NEXT]
+++++++++++++++++++++++++++++++++++++++++++++++++++
In my view, as an information specialist, the two words that stand out for me are: **training** and **cardiotocography.**

That isn't to say the rest are not important or relevant, but for this task, which seeks to make a judgment on records at title and abstract level, and not mistakenly throw anything out that *might* be relevant, it's safest to keep the inclusion criteria quite broad.

## Health professional training for cardiotocography interpretation and management

[NEXT]

++++++++++++++++++++++++++++++++++++
For this task you'll be presented records that will look a bit like this:

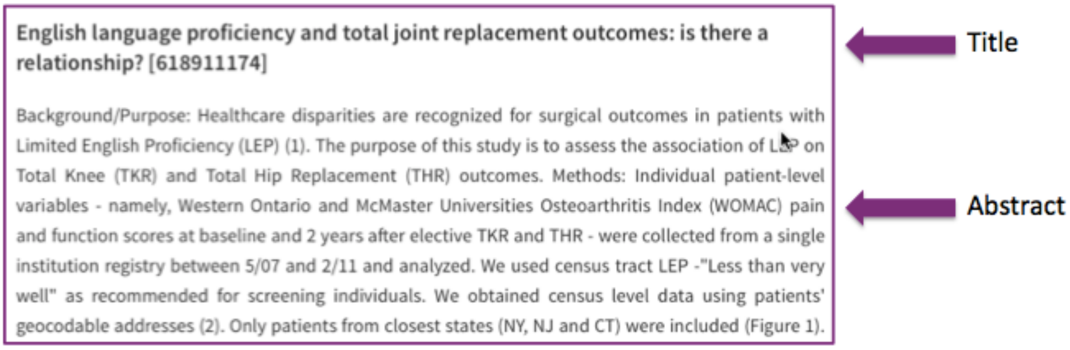
**English language proficiency and total joint replacement outcomes: is there a relationship? [618911174]**

Background/Purpose: Healthcare disparities are recognized for surgical outcomes in patients with Limited English Proficiency (LEP) (1). The purpose of this study is to assess the association of LEP on Total Knee (TKR) and Total Hip Replacement (THR) outcomes. Methods: Individual patient-level variables - namely, Western Ontario and McMaster Universities Osteoarthritis Index (WOMAC) pain and function scores at baseline and 2 years after elective TKR and THR - were collected from a single institution registry between 5/07 and 2/11 and analyzed. We used census tract LEP -"Less than very well" as recommended for screening individuals. We obtained census level data using patients' geocodable addresses (2). Only patients from closest states (NY, NJ and CT) were included (Figure 1).


They will all have a title, and the vast majority will have an abstract as well.

[NEXT]
++++++++++++++++++++++++++++++++++++

For each record you'll need to make a decision from a choice of three options:

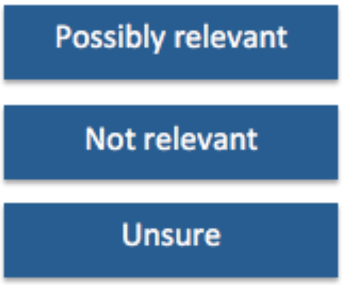


Classify records as ***Possibly relevant*** if they are about training AND cardiotocography.

Classify records as ***Not relevant*** if they are not about training or cardiotocography.

When you aren't sure, simply select ***Unsure***.

+++++++++++++++++++++++++++++++++++++
So, what does the term 'training' mean for this review?

It means educational activities aimed at healthcare professionals that will include one of the following:

- Improve/refresh existing knowledge
- Learn new skills
- Change behaviours/attitudes of health professionals

The delivery of educational activities could take a wide range of formats such as lectures, workshops, online learning courses, or courses aimed at training whole teams:

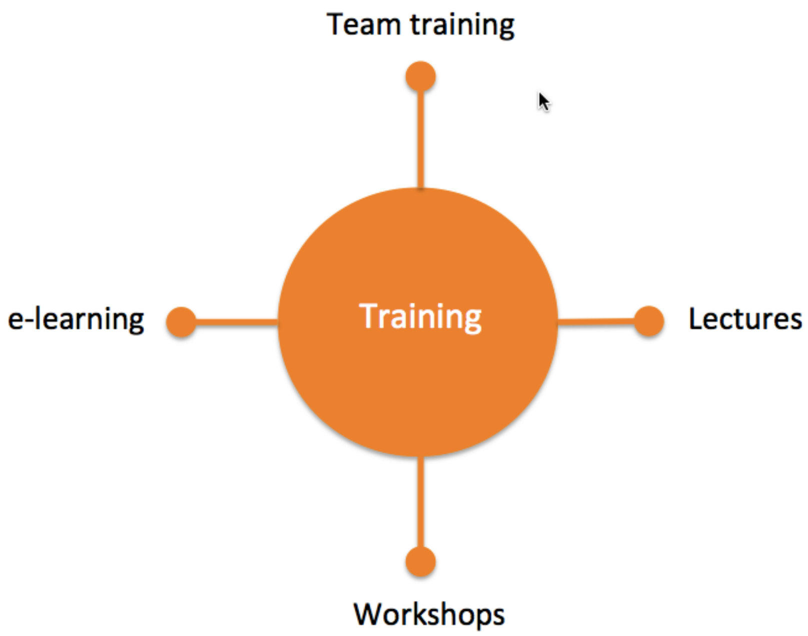


[NEXT]
+++++++++++++++++++++++++++++++++++++++++++
What is cardiotocography monitoring?

Cardiotocography monitoring is a type of foetal heart rate monitoring. It is used around the world, and most often for high-risk pregnancies. There are other types of foetal heart rate monitoring but we are specifically interested in cardiotocography.

This type of monitoring measures both the foetus's heart rate and the woman's uterine contractions.

[NEXT]
+++++++++++++++++++++++++++++++++++++++++++
Records need to contain both elements. Records that are about some kind of training for something other than cardiotocography are not relevant.

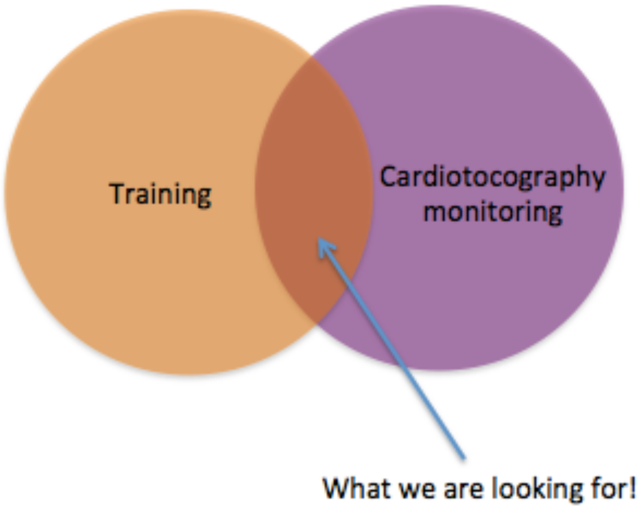

I think it’s time to try a few.
Ten practice records coming up and then you’re done!

[NEXT]
+++++++++++++++++++++++++++++++++++++++++++++++++++++

[Load text file 1 made up of 10 records]

Record 1: 8869534 (PMID); 127188734 (Accession number)
***Computer versus lecture: strategies for teaching fetal monitoring***
*The purpose of this study was to determine how well novice nurses learned basic fetal monitoring concepts from a computer-assisted instructional program and a scripted lecture with equivalent content. A pretest-posttest experimental design was conducted. Forty-eight junior baccalaureate nursing students beginning their first maternity rotation were recruited for the study from a southwestern university. Thirty-nine students provided complete data sets. None had prior experience or education in fetal monitoring. Two strategies were used to teach basic fetal monitoring concepts: a scripted lecture with black-and-white transparencies and an equivalent computer-assisted instructional program. Statistical significance was set at p = 0.05. There were no significant differences between the computer and lecture groups' learning on the basis of age, education, or vision. There was a positive, nonsignificant gain in mean scores from the pretest to the posttest for both groups. The greatest difference was in the program completion time with the computer-assisted instruction group completing the program 43.6% faster than the lecture group.*

QT: Does this record look like it might be relevant?
QO: Possibly relevant // Not relevant // Unsure
QA: Possibly relevant
AC: This record does look possibly relevant. The title, whilst it doesn’t actually use the words training or cardiotocography, it does mention two training or learning methods – computer versus lecture – and is about foetal monitoring, of which one technique is cardiotocography monitoring. The abstract goes into more detail about the training methods and whilst cardiotocography still doesn’t get mentioned explicitly, we would want to keep this record. Let’s try another one.

Record 2: 8656304 (PMID); 126226362 (Accession number)
***Evaluation of a fetal monitoring education program***
*OBJECTIVE: To evaluate the effectiveness of a fetal monitoring education program in increasing nurses' knowledge and clinical skills. DESIGN: Multicenter randomized control trial. SETTING: Twelve hospitals in eastern Ontario, Canada. PARTICIPANTS: One hundred nine volunteer registered nurses randomly assigned, within each hospital, to an experimental (n = 47) or control (n = 62) group. Ninety-six nurses (40 in the experimental group and 56 in the control group) completed the 6-month follow-up (88% retention). INTERVENTIONS: The experimental group participated in a 1-day fetal monitoring workshop and a review session 6 months later. MAIN OUTCOME MEASURES: Performance on a 45-item knowledge test and a 25-item skills checklist. The passing score was at least 75% correct on each test. RESULTS: The percentage of nurses in the experimental group passing both the knowledge and the clinical skills tests after the workshop was significantly higher (p < 0.01) than that of the nurses in the control group: 68.1% versus 6.5%, respectively. A large difference between the groups remained at the 6-month follow-up (experimental, 45%; control, 6.5%). The performance of the nurses in the experimental group improved to an 85% pass rate after they attended the 6-month review session. CONCLUSION: This comprehensive, research-based program is effective in increasing fetal monitoring knowledge and clinical skills.*

QT: Does this record look like it might be relevant?

QO: Possibly relevant // Not relevant // Unsure
QA: Possibly relevant
AC: This is quite similar to the last record. It is possible relevant despite the fact it also does not specifically mention cardiotocography. So we're learning that just because a record doesn't contain the specific kind of foetal heart monitoring we are concerned with for this review, it doesn't mean the record is not relevant. Time for another one.

Record 3: 21295386 (PMID); 51260043 (Accession number)
***Midwives' and doctors' attitudes towards the use of the cardiotocograph machine***
*The appropriate use of the cardiotocograph (CTG) machine in the clinical setting is an issue of concern for midwives and doctors. Objective: to examine midwives and doctors attitudes towards the use of the CTG machine in labour ward practice. Background: this small study provides new insight into the attitudes of doctor and midwives towards the use of CTG. Design: an exploratory descriptive design that used a combination of qualitative and quantitative approaches. A valid and reliable tool designed by Sinclair (2001) was used to measure the attitudes of doctors and midwives towards CTG usage. In addition, follow-up semi-structured interviews with doctors and midwives were conducted. Setting: a maternity unit in Northern Ireland. Participants: participants selected had worked in the labour ward within the last year (n=56 midwives; n=19 doctors). Six midwives and two doctors were randomly selected to participate in the interviews. Findings: the study demonstrated a favourable disposition towards the use of CTG machines with 72.5% (n=29) of respondents indicating that they viewed CTG technology positively and 87.5% (n=25) indicating they were confident about their skill in interpreting CTG tracings. The majority of the respondents (60.0%, n=24) felt that their training adequately prepared them for using CTGs. The illustrative accounts provided by the respondents demonstrated a predominant belief that CTG technology continues to have a role in monitoring and detecting abnormalities in the fetal heart rate but this role is limited by how well the CTG is used and interpreted. The interviews confirmed the data obtained from the questionnaires and revealed a number of professional needs and concerns relating to CTG usage. Implications for practice: the implication of this study may be focused on addressing the training needs of students, newly qualified staff and regular updates for employed staff. There was some concern that this technology may be deskilling staff and therefore there is a need to improve confidence levels in using alternatives to this type of fetal monitoring.*

QT: Does this record look like it might be relevant?
QO: Possibly relevant // Not relevant // Unsure
QA: Not relevant
AC: This record, despite being about the cardiotocography machine isn't relevant because there is no training aspect to it. It's describing an article about midwives and doctors attitudes. We need both elements: the training component and the foetal heart monitoring, to be present. Let's try one that might be quite tricky!

Record 4: 11408301 (PMID); 32575410 (Accession number); 2001224366 (Embase accession number)
***Randomised controlled trial of cardiotocography versus Doppler auscultation of fetal heart at admission in labour in low risk obstetric population.***
*Objective: To compare the effect of admission cardiotocography and Doppler auscultation of the fetal heart on neonatal outcome and levels of obstetric intervention in a low risk obstetric population. Design: Randomised controlled trial. Setting: Obstetric unit of teaching hospital. Participants: Pregnant women who had no obstetric complications that warranted*

*continuous monitoring of fetal heart rate in labour. Intervention: Women were randomised to receive either cardiotocography or Doppler auscultation of the fetal heart when they were admitted in spontaneous uncomplicated labour. Main outcome measures: The primary outcome measure was umbilical arterial metabolic acidosis. Secondary outcome measures included other measures of condition at birth and obstetric intervention. Results: There were no significant differences in the incidence of metabolic acidosis or any other measure of neonatal outcome among women who remained at low risk when they were admitted in labour. However, compared with women who received Doppler auscultation, women who had admission cardiotocography were significantly more likely to have continuous fetal heart rate monitoring in labour (odds ratio 1.49, 95% confidence interval 1.26 to 1.76), augmentation of labour (1.26, 1.02 to 1.56), epidural analgesia (1.33, 1.10 to 1.61), and operative delivery (1.36, 1.12 to 1.65). Conclusions: Compared with Doppler auscultation of the fetal heart, admission cardiotocography does not benefit neonatal outcome in low risk women. Its use results in increased obstetric intervention, including operative delivery.*

QT: Does this record look like it might be relevant?
QO: Possibly relevant // Not relevant // Unsure
QA: Not relevant
AC: This record is about cardiotocography but it isn't about training health professionals. It's a randomized trial that compared two different ways of measuring foetal heart rates in a low risk population. For previous Cochrane Crowd tasks we're often on the hunt for randomised trials. However, this task is different. Here we're looking for records based on some topic criteria: some sort of training method AND cardiotocography monitoring. You are almost at the halfway mark!

Record 5: 16876921 (PMID); 44755202 (Accession number)
***A randomised-controlled trial evaluating a fetal monitoring education programme***
*Objective: to evaluate the effectiveness of a fetal monitoring education programme on midwives' fetal monitoring knowledge and intrapartum cardiotocograph interpretation skills. Design: two group, before-after, randomised-controlled trial. Setting: two maternity hospitals in the Republic of Ireland. Participants: 55 midwives were randomly assigned to either the experimental group (n=27) or the control group (n=28). Interventions: the experimental group participated in a 1.5 hr fetal monitoring education programme, whereas the control group attended an alternative education programme consisting of a non-fetal-monitoring-related video presentation. Measurements: the primary outcomes of interest were fetal monitoring knowledge and intrapartum cardiotocograph interpretation skills test. Findings: in the fetal monitoring knowledge post-test, the median percentage correct responses for the control and experimental groups were 56% (IQR 18.75) and 88% (IQR 12.5), respectively. This difference, 31.2%, was statistically significant (U=78.5, 95.1% CI -31.25 to -18.75, p<0.001). In the intrapartum cardiotocograph interpretation skills post-test, the median percentage correct responses for the control and experimental groups were 55.6% (IQR 16.7) and 66.7% (IQR 22.2), respectively. This difference, of 11.1%, was statistically significant (U=186, 95.2% CI -16.67 to -5.56, p<0.001). Key conclusions and implications for practice: attendance at a short (1.5 hr), in-service fetal monitoring education programme can increase midwives' fetal monitoring knowledge and cardiotocograph interpretation skills. The availability of in-service fetal monitoring education programmes is sporadic, often less than recommended, and is a cause for concern.*

QT: Does this record look like it might be relevant?
QO: Possibly relevant // Not relevant // Unsure
QA: Possibly relevant

AC: Okay, so we just had a record that was describing a randomized trial and we classified it as “not relevant”. Now, we have another record describing a randomised trial that is relevant. Why is this one relevant? Because it is a study looking to evaluate an educational program about foetal monitoring. Just what we’re looking for! Only five more records to go.

Record 6: 251739 (PMID); 8374326 (Accession number); 1978378728 (Embase accession number)
***A statewide program to teach nurses the use of fetal monitors***

QT: Does this record look like it might be relevant?
QO: Possibly relevant // Not relevant // Unsure
QA: Unsure
AC: Sometimes there is no right answer, only a wrong one. Here we were given only a title to work with. It can be harder to make a firm decision from a title. If you said either Possibly relevant or Unsure, then you did well. The title is promising as it appears to be about teaching nurses how to use foetal monitors. We would want to keep this record and try to get hold of the full article in order to make a final decision on whether it is for inclusion within the review.

Record 7: 21658193 (PMID); 362051693 (Accession number); 2011360643 (Embase accession number)
***Evaluation and impact of cardiotocography training programmes: A systematic review***
*Background: The interpretation and management of cardiotocography (CTG) tracings are often criticised in obstetric malpractice cases. As a consequence, regular CTG training has been recommended, even though little is known about the effect of CTG training. Objectives: To perform a systematic review of the existing literature on studies on CTG training in order to assess educational strategies, evaluation of training programmes, and impact of training programmes. Search strategy: The Medline database was searched to identify studies describing and/or evaluating CTG training programmes. The literature search resulted in 409 citations. Selection criteria: Twenty studies describing and evaluating CTG training programmes were included. There was no restriction on study design. Data collection and analysis: Data regarding study design, study quality, educational strategies used for training in CTG interpretation and decision making, target groups, number of participants, methods used for evaluation, quality of evaluation, level of evaluation and results of training was extracted from 20 articles, and analysed using Kirkpatrick's four-level model for the evaluation of education. Main results: Training was associated with improvements on all Kirkpatrick levels, resulting in increased CTG knowledge and interpretive skills, higher interobserver agreement, better management of intrapartum CTG, and improved quality of care. Computer-based training (CBT) might be less time-consuming than classroom teaching. Clinical skills seem to decrease faster than theoretical knowledge. Author's conclusions: Training can improve CTG competence and clinical practise. Further research on CBT, test-enhanced learning and long-term retention, evaluation of training and impact on clinical outcomes is recommended.*

QT: Does this record look like it might be relevant?
QO: Possibly relevant // Not relevant // Unsure
QA: Possibly relevant
AC: In other Cochrane Crowd tasks you might be used to rejecting systematic reviews but for this task, if they are on topic, then keep it in. The review is indeed all about evaluating training programmes on cardiotocography.

Record 8: 6615739 (PMID); 1983230847 (Embase accession number); 13052004 (Accession number)

***Fetal scalp blood lactate as an indicator of intrapartum hypoxia***

*Fetal scalp blood lactate was measured during labour by a simple, rapid method and its value as an indicator of fetal intrauterine hypoxia was assessed and compared with that of pH measurement. The normal ranges of lactate concentrations and of pH values were calculated. Significantly higher concentrations of lactate and lower pH values were found in samples of scalp blood taken close to delivery from babies with Apgar scores of <= 6 at 1 min compared with those from healthy babies with Apgar scores of <= 7 at 1 min. A similarly significant difference was observed between the cord blood lactate and pH values of these two groups of babies. Ominous fetal heart rate patterns were associated with higher lactate concentrations and lower pH values in fetal scalp blood than were normal fetal heart rate patterns. The measurement of fetal scalp blood lactate or pH, or continuous electronic fetal heart rate monitoring were equally good at predicting the condition of the infant at birth.*

QT: Does this record look like it might be relevant?
QO: Possibly relevant // Not relevant // Unsure
QA: Not relevant
AC: This record is not relevant. It's a real example from the search results that have been retrieved from the search process so it's a good example of a record to be rejected. It's likely that a good proportion of records won't be relevant.

Record 9: 19915414 (PMID); 358083229 (Accession number)

***The Fetal heart rate collaborative practice project: Situational awareness in electronic fetal monitoring- A Kaiser Permanente perinatal patient safety program initiative***

BACKGROUND: Electronic fetal monitoring has historically been interpreted with wide variation between and within disciplines on the obstetric healthcare team. This leads to inconsistent decision making in response to tracing interpretation. PURPOSE: To implement a multidisciplinary electronic fetal monitoring training program, utilizing the best evidence available, enabling standardization of fetal heart rate interpretation to promote patient safety. METHODS: Local multidisciplinary expertise along with an outside consultant collaborated over a series of meetings to create a multimedia instructional electronic fetal monitoring training program. After production was complete, a series of conferences attended by nurses, certified nurse midwives, and physician champions, from each hospital, attended to learn how to facilitate training at their own perinatal units. All healthcare personnel across the Kaiser Permanente perinatal program were trained in NICHD nomenclature, emergency response, interpretation guidelines, and how to create local collaborative practice agreements. Metrics for program effectiveness were measured through program evaluations from attendees, the Safety Attitudes Questionnaire. RESULTS: Program evaluations rendered very positive scores from both physicians and clinicians. Comparing baseline to 4 years later, the perception of safety from the staff has increased over 10% in 5 out of the 6 factors analyzed. SUMMARY: Active participation from all disciplines in this training series has highlighted the importance of teamwork and communication. The Fetal Heart Rate Collaborative Practice Project continues to evolve utilizing other educational modalities, such as online EFM education and unit-based interdisciplinary tracing reviews. Copyright © 2009 Wolters Kluwer Health.

QT: Does this record look like it might be relevant?
QO: Possibly relevant // Not relevant // Unsure
QA: Possibly relevant

AC: This record should be given a ‘possibly relevant’ classification. Under Purpose it says: “To implement a multidisciplinary electronic fetal monitoring training program”. Right, last practice record coming up!

Record 10: 10379125 (PMID); 29195097 (Accession number); 1999148298 (Embase accession number)
***A randomized controlled trial comparing midwife-managed care and obstetrician-managed care for women assessed to be at low risk in the initial intrapartum period.***
*Objective: To compare the efficacy of midwife-managed care and obstetrician-managed care for women assessed to be at low risk in the initial intrapartum period. Methods: 1050 women assessed to be at low risk on admission to labour ward in the Prince of Wales Hospital participated in this study. By computer-generated random allocation, 563 (54%) women were assigned to Group A (experimental) under midwifery care, and 487 (46%) women to Group B (control) under obstetrician care. The outcomes and complications between the 2 groups were compared. Data were analyzed by 2 x 2 contingency tables and Chi-square. Results: 150 (26.6%) women in the experimental group were taken over by the obstetricians. 46 (30.7%) women were transferred to obstetrician-management for the preference of epidural analgesia. The other reasons for taken over the remaining 104 (69.3%) women were fetal distress, poor progress of labour, complications in first or second stage of labour. The experimental group had less oxytocic augmentation (Chi-square = 7.49, p = 0.006) and the insertion of intravenous infusion (Chi-square = 5.34, p = 0.02). Both groups had similar outcomes on normal delivery, operative vaginal delivery, caesarean section and complications. Conclusions: Midwife-managed care is as safe as obstetrician-managed care for women who were assessed to be at low risk in the intrapartum period. Routine visit by obstetrician is not necessary and the midwives are able to detect complications in the course of labour and alert the obstetrician for taking the necessary action.*

QT: Does this record look like it might be relevant?
QO: Possibly relevant // Not relevant // Unsure
QA: Not relevant
AC: This record should be classified as *Not relevant.* It’s a randomised trial comparing two modes of care of women in labour. It’s not therefore about training for cardiotocography. That’s it! You are done. Time to grab a cup of tea before diving into some live records?

[NEXT]
