# Supplementary material for: Crowdsourcing citation-screening in a mixed-studies systematic review: a feasibility study
Source: BMC Med Res Methodol. 2021 Apr 26;21:88. doi: 10.1186/s12874-021-01271-4 (PMC8077753; doi:10.1186/s12874-021-01271-4)
Supplement: Supplementary file 4 — Additional file 4. [file 12874_2021_1271_MOESM4_ESM.pdf]

Cochrane Crowd and THIS Institute Survey
May 2018

Question 1: Which of the following describes you (tick all that apply)?
- I'm a student in a healthcare related area
- I'm a student in a non-healthcare related area
- I work in a healthcare related area
- I teach/train in a healthcare related area
- I'm a patient
- I'm a carer
- I'm retired
- I'm not any of these

Question 2: What was main interest or motivation in signing up for this task? Please select all that apply and rank them in order of main motivation to lesser motivation/s.
- Interest in this area of research
- The chance to get acknowledgement in a review
- The chance to get a certificate of involvement
- To do something good for a well-respected organisation
- It sounded fun/interesting
- Interest in systematic review production methods

Question 3: Overall, did you enjoy it?
- Yes
- No

Question 4: How did you find it to screen abstracts for a particular topic?
- Very easy
- Easy
- Neutral – neither easy nor difficult
- Difficult
- Very difficult

Question 5: Did you find this task more or less interesting than Cochrane Crowd's standard RCT identification task?
- Less interesting
- About the same
- More interesting
- Unsure

Question 6: Did you find the highlighted words and phrases useful?
- Yes
- No

Question 7: Do you think the highlighted words and phrases influenced your decisions?

- Yes
- No

Question 8: What, if anything, would have improved the overall experience?

Question 9: Finally, a huge thank you for taking part and for completing this survey. Are there any other comments you'd like to make about the pilot task and how it went?
